# A dataset of clinically generated visual questions and answers about radiology images

**DOI:** 10.1038/sdata.2018.251

**Published:** 2018-11-20

**Authors:** Jason J. Lau, Soumya Gayen, Asma Ben Abacha, Dina Demner-Fushman

**Affiliations:** 1 Lister Hill National Center for Biomedical Communications, National Library of Medicine, Bethesda, MD, USA

**Keywords:** Data mining, Radiography

## Abstract

Radiology images are an essential part of clinical decision making and population screening, e.g., for cancer. Automated systems could help clinicians cope with large amounts of images by answering questions about the image contents. An emerging area of artificial intelligence, Visual Question Answering (VQA) in the medical domain explores approaches to this form of clinical decision support. Success of such machine learning tools hinges on availability and design of collections composed of medical images augmented with question-answer pairs directed at the content of the image. We introduce VQA-RAD, the first manually constructed dataset where clinicians asked naturally occurring questions about radiology images and provided reference answers. Manual categorization of images and questions provides insight into clinically relevant tasks and the natural language to phrase them. Evaluating with well-known algorithms, we demonstrate the rich quality of this dataset over other automatically constructed ones. We propose VQA-RAD to encourage the community to design VQA tools with the goals of improving patient care.

## Background & Summary

Visual question answering (VQA) is a computer vision and artificial intelligence (AI) problem that aims to answer questions about images. As more of medicine is digitized and medical data continues to grow, there is enormous opportunity for multimodal tools such as VQA to benefit patients, clinicians, and researchers. Radiology, with its wealth of images and textual reports, is a prime area where VQA could assist radiologists in reporting findings for a complicated patient or benefit trainees who have questions about the size of a mass or presence of a fracture. Many different techniques are applied to build VQA systems including computer vision, natural language processing, and deep learning. These systems need to be trained for the task and evaluated on large data collections consisting of images and pairs of questions asked about the images with corresponding answers. Although there has been great progress in image recognition in radiology^1^, the datasets that allowed this are not quite generalizable to VQA because none of the datasets have question-answer pairs directed at the images^2,3^. Deep learning models require vast amounts of data to approach abilities of humans and as a result, the most popular training datasets are crowdsourced with general public knowledge^4–8^ or synthetically generated^9,10^. The images in most of these datasets were taken from Microsoft Common Objects in Context (MSCOCO)^11^. While researchers have tried balancing these large datasets^5,12^, bias in selection of images and questions makes it difficult to transfer models trained on these datasets to specific applications. For example, the creators of VizWiz^13^, the first VQA dataset designed from images taken by blind users and visual questions from those use cases, demonstrated challenges in using the state-of-the-art models trained on the larger VQA datasets to predict answers from their VizWiz blind users generated data.

While visual questions are prevalent in medicine, most communications between clinicians are not usually documented in the patient’s record or available for researchers. Medical education is one area where collections of visual questions exist. Through their training and continued medical education, clinicians will take exams testing their knowledge of medical images. However, these test questions often go beyond the scope of the image and require extra knowledge about epidemiology or next step treatments. Most exam questions may not be suitable for teaching machines to answer descriptive questions about images, but they still have a role for more abstract machine assisted question answering in the future.

In 2018, ImageCLEF-Med released a radiology dataset^14^ and coordinated the first community-wide VQA challenge in medicine. While the dataset is an excellent starting point for the medical domain, several design issues prevent useful clinical applications. To overcome the lack of readily available natural visual questions, questions and answers were automatically generated from corresponding captions. Unfortunately, this resulted in many artificial questions that do not always make sense, to the point where a human could not reason what the questions were trying to ask. Another issue with the dataset is that images were automatically captured from PubMed Central articles. This automation may not help VQA support clinical tasks as the dataset included composites of a series of images and 3D reconstructions that are rarely useful for direct patient care. If VQA tools are to assist in clinical processes, the datasets need to be designed with aligned goals.

**Figure 1:**
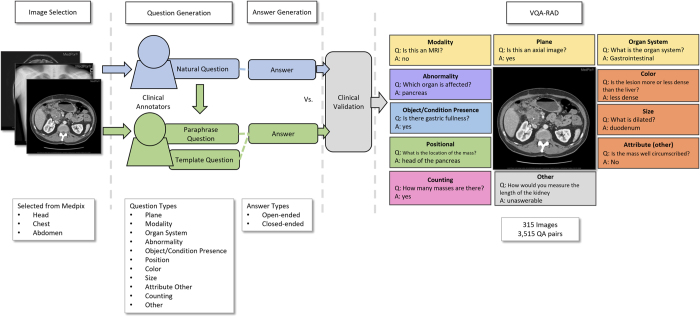
Flow Diagram of VQA-RAD build.

We introduce VQA-RAD, a manually constructed VQA dataset in radiology where questions and answers about images are naturally created and validated by clinicians. Trading off quantity of automatic generation, VQA-RAD is a high-quality design with only 60 hours of specialist contributions. Structured like other existing VQA datasets, each question can be answered with a single image alone. The images are a balanced sample from MedPix®, an open-access radiology archive of case reports and teaching cases. These images were presented to clinicians who wrote unguided questions they would ask a colleague or radiologist. To further explore the natural clinical language, clinicians also paraphrased questions in both free-form and template structures. All question-answer pairs are manually validated and categorized, helping to characterize clinically important areas of focus. The flow of building the VQA-RAD dataset is shown in Fig. 1. We demonstrate the value of VQA-RAD and use cases by applying several well-known algorithms.

## Methods

### Image Selection

We sampled images from teaching cases in MedPix, https://medpix.nlm.nih.gov/, an open-access database of radiology images and teaching cases. Our sampling criteria were as follows: (1) Only one image for each teaching case, so that all images represented unique patients. (2) All images are sharp enough to identify individual structures. (3) Images are clean of radiology markings, such as arrows or circles. (4) Images have captions that correspond to the image and are detailed enough to describe at least one structure.

Today, diagnostic imaging often contains stacks of images, which is a challenge that VQA tools will need to incorporate to be useful for assisting radiology. For a dataset just emerging into medicine, we wanted to start with simple one image and one question pairs, enabling comparisons with current existing VQA dataset structures and algorithms. To reduce bias of having multiple images of the same pathology, we chose to limit one image per case, since each case is a unique patient. In addition, MedPix images are stored in JPG format, which needs to be taken into consideration when using the images for teaching and for training AI approaches.

Captions include plane, modality, and image findings that were generated and reviewed by expert radiologists. In total, we selected 104 head axial single-slice CTs or MRIs, 107 chest x-rays, and 104 abdominal axial CTs. The balanced distribution from head, chest, and abdomen should help determine if visual questions differ for each organ system and if the algorithms perform differently on different regions.

### Question Type and Answer Type Generation

In addition to categorizing images, we define several question and answer types. Broad question types were initially developed from a combination of using Task Directed Image Understanding Challenge (TDIUC) categories^12^, annotating radiology reports from MedPix, and categorizing CME questions from open-access domains such as MedPix, AuntMinnie (https://www.auntminnie.com/), and Radiopaedia.org (https://radiopaedia.org/). Question types were further refined after analyzing an initial sample of visual questions from annotators. The final taxonomy of medical VQA in our dataset is shown in Table 1.

### Annotators

The tasks of assisting clinicians with radiology are different and range from support for those just learning the basics of reading a plain film x-ray, to those who are senior radiologists investigating rare presentations of diseases. We selected medical students and fellows who had at least their core clinical rotations completed because they will better understand how radiology is incorporated into every day clinical decision making and patient care. These trainees’ questions and tasks are closer to those of attending trainees, compared to students just starting medical school. However, we do expect that there will be additional needs from resident trainees as their personal skill in reading radiology images improves and they have more nuanced questions. For example, a junior trainee may ask if there is presence of a pneumothorax, while a senior, who can already recognize air in the lungs, may ask more questions about the size of the pneumothorax since this has implications for invasive treatment.

Questions and answers were generated by 15 volunteer clinical trainees using a web-interface developed for collecting the questions and the answers. We asked for volunteers from the clinical fellows from the NIH and students from class of 2018/2019 University of Massachusetts, classmates of one of the authors, Jason Lau. All participants had completed the core rotations of medical school, which typically occurs during the 3^rd^ year of school and exposes students to major fields of medicine such as surgery, internal medicine, neurology, etc. This ensures that all participants have basic clinical radiology reading skills and were exposed to a variety of settings where radiology was vital to the management of patients. Our participants had training from different regions of the U.S. and there was one board certified pathologist, three applying into ophthalmology, four applying into family medicine, two applying into internal medicine, two applying into emergency medicine, one applying into radiology, one applying into orthopedics, and one dermatology.

### Question and Answer Generation

Participants enerated questions and answers in a two-part evaluation (shown in Fig. 1) from December 2017 to April 2018. Each participant reviewed at least 40 randomized images. For the first 20 images, participants provided “free-form” questions and answers without any restrictions. We instructed participants to create “free-form” question about the images by phrasing them in a natural way as if they are asking a colleague or another physician. The image alone had to be sufficient to answer the question and there should only be a single correct answer. We asked that answers to the visual questions be based off their level of knowledge. Since many of the participants were still in medical training, we provided captions with some image findings, plane, and modality information to provide additional ground truth reassurance.

For the next 20 images, participants were randomly paired and given another participant’s images and questions. They were asked to generate “rephrased” and “framed” questions based off the given “free-form” questions with corresponding image and caption. We asked the participants to paraphrase the question in a natural way and generate an answer that agreed with both the original and the paraphrased questions.

Participants generated “framed” questions by finding the closest question structure from a list of templates and filling in the blank spaces to retain the answer to the original questions.

### QuestionAnswer and Question Type Validation

After completion of the evaluations, we used several methods to validate questions answer pairs and question types. During the paraphrasing part of the evaluation, participants answered another person’s questions. The answers could have strict or loose agreement. We defined strict agreement when the question and answer format and topic were the same. In loose agreement, the topic of the questions is the same or similar even though the answers may differ. Three subcategories of loose agreement are defined: inversion, conversion, and subsuming.

Examples of each as follows:

**Inversion:** Q1: “Are there abnormal findings in the lower lung fields?” is a negation of Q2: “Are the lower lung fields normal?”

**Conversion**: Q1: “How would you describe the abnormalities?” is open-ended while Q2: “Are the lesions ring-enhancing?” is closed-ended

**Subsumption:** Q1: “Is the heart seen in the image?” subsumes Q2: “is the heart seen on the left?”

We calculate F1 scores of user agreement for questions considered ‘evaluated’, meaning they were reviewed by two annotators. Disagreements amongst question category types were reviewed at weekly team meetings to determine general rules for all content. Disagreements in objective findings of images were reviewed by using MedPix metadata and advice from expert radiologist to find ground truth. This in-depth review was only required for 280 question-answer pairs. Questions are labeled as ‘not evaluated’ if they are not reviewed by a second participant or the paraphrased question is not similar enough to be used as validation. Both the evaluated and not evaluated questions are used as part of the test and training set.

We validated question types assigned by the participants. Final categorization was determined through consensus with the research team to resolve disagreements.

We anticipate that a VQA-RAD pipeline is extensible to produce more test data and training data for traditional machine learning approaches. We believe that more training data sufficient for training deep learning approaches could be produced automatically, bootstrapping from the VQA-RAD collection and using recent approaches to image synthesis and data augmentation^15^.

## Data Records

The full dataset is archived with Open Science Framework (Data Citation 1). All question-answer pairs referencing images are stored in a single dataset that we provide in three common formats: JSON, XML, and Excel. The dataset contains 14 variables of unique identifiers and categorization with a total of 2,248 elements. The 315 corresponding radiological images are contained in a separate folder. Naming conventions and descriptions of all files can be found in a provided Readme file.

## Technical Validation

### Analysis of Questions and Answers

The final VQA-RAD dataset contains 3,515 total visual questions. Of these, 1,515 (43.1%) are free-form. 733 questions are rephrased which means 48.3% of the free-form questions have corresponding paraphrasing. The remaining 1,267 questions are framed and have corresponding free-form and rephrased questions. On average, there are 10 questions per image.

Of the free-form and rephrased questions, 75% (1,691) are evaluated using seven pairs of annotators. User agreement F1-scores range from 0.78 to 0.95 with a mean of 0.85.

All questions are short, with medians, mean, and modes ranging from 5 to 7 words per question. After converting all words to lowercase and comparing sentences for unique structures, the free-form and rephrased questions have more distinct questions (87% and 93% questions are distinct) compared to framed questions which had 49% uniquely structured questions. Answers are also short, with median and modes of 1 word per question (mean of 1.6). Comparing all lowercase answers for unique structures, we observed 486 distinct answers. This represents 32% distinct answers of the total 1,515, which we expect because about half of all answers are either ‘yes’ or ‘no’.

Subgroup analysis of free-form questions demonstrates the relationship between question types and answer types. Of the 11 question types, Presence questions dominate, with 36% (483) of free-form questions. The least frequent question type is Counting, with only 1% (15) questions. The QA pairs are split into 42% (637) open-ended answer types and 58% (878) close-ended. Yes/no questions represent 92% of the close-ended QA pairs, and ‘yes’ (405) is as frequent as ‘no’ (406.)

**Figure 2:**
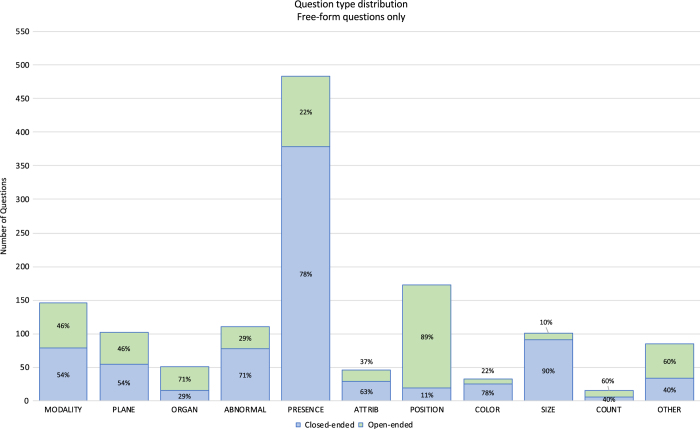
Breakdown of different types of Closed vs Open-ended free form questions shows that certain question types are more likely to be open-ended: positional, counting questions and other.

Subgrouping question and answer types, we observe that people think about certain categories differently (See Fig. 2). Abnormalities, Presence, and Size questions are mostly closed-ended questions. The majority of Organ system and Positional questions are open-ended. While these imbalances are important for learning how to better balance future datasets to ensure that algorithms have enough data to learn to predict answers, these observations suggest that different approaches may be required for different question types. The favoring distribution of closed-ended size questions may suggest that in clinical practice, asking questions about whether something is “enlarged” or “atrophied” in reference to an acceptable relative size is more useful than asking an open-ended “what is the size of the organ?”.

### Relationship of Closed and Open Questions

We next analyzed the types of words for each question type (The full analysis is provided with the dataset distribution). For each question category, we tokenized the words and listed the top 10 most common distinct words. Many of these words are unique to the question category and can be used as keywords to help guide question categorization. The frequencies of words give insight into the context in which certain questions are likely to be asked. For example, ‘heart’, ‘cardiac’, ‘cardiomegaly’, and ‘dilated’ are frequent words in the size questions, which suggests that participants thought about size of the heart more commonly than other organs.

### Relationship of Image Type and Organ System

Examining the question categories in relation to image type, we observed several differences in what clinicians ask when they see images of the head, chest, or abdomen. Presence questions are asked evenly about abdomen and chest, with fewer head images, although remaining the most commonly asked question type. Positional and Color questions were more prevalent with images of the head. Contrastingly, positional questions were less frequent with abdominal images. Organs in the head, in particular structures of the brain, are much more fixed in location compared to organs in the abdomen. Therefore, positional questions are more relevant in the head where one can reference locations. In the abdomen, asking if something is seen in the image is more clinically relevant or easier to determine than knowing the location. Another trend is that size questions are asked most frequently in chest images, agreeing with the distinct words we described earlier. The relationship between question type and image type gives some insight into the patterns of clinical relevancy that clinicians may have. Not all questions are useful and maybe shouldn’t be asked for every image.

### VQA-RAD Benchmarking

We validated the dataset by evaluating the performance of well-known VQA algorithms and their accuracy at answering visual questions from the VQA-RAD dataset.

### Baselines

We include two well-known VQA methods: Multimodal Compact Bilinear pooling (MCB)^16^ and Stacked Attention Network (SAN)^17^. The MCB model, winner of the 2016 VQA challenge, uses a multimodal compact bilinear pooling method that first predicts attention visually and textually then combines these features with question representation. MCB includes three components: a CNN image model, an LSTM question model, and MCB pooling that first predicts the spatial attention and then combines the attention representation with the textual representation to predict the answers. For the image model, we used ResNet-152 pre-trained on imageNet. For the question model, a 2-layer LSTM model was used.

The SAN model is a stacked attention model that queries images multiple times to progressively narrow an attention. Similarly to MCB, SAN includes three components: the image model based on a CNN to extract high level image representations, the question model using an LSTM to extract a semantic vector of the question and the stacked attention model which locates the image regions that are relevant to answer the question. We used the last pooling layer of VGG-16 pre-trained on imageNet as image features, and the last LSTM layer as question features. The image features and the question vector were used to generate the attention distribution over the regions of the image.

The MCB and SAN were pre-trained on ResNet^18^ and VGGNet^19^ respectively to extract image features and then both trained on VQA V1.0 dataset training and validation^4^. We refer to these baseline models as MCB-VQA1.0 and SAN-VQA1.0, when trained on VQA V1.0. We then trained the models on the ImageCLEF-VQA-Med^14^ training set to see how an existing medical VQA dataset influences the models. We refer to the ImageCLEF trained models as MCB-CLEF and SAN-CLEF. We then train the two networks with VQA-RAD training set creating MCB-RAD and SAN-RAD. Since VQA-RAD is a small dataset, we combined ImageCLEF-VQA-Med with VQA-RAD and trained the networks to explore any synergistic effects. As part of a baseline reference for closed-ended questions, we include predicted answer sets of all ‘yes’ and ‘no’.

### Metrics

From VQA-RAD we separated out a training set and test set. VQA-RAD test set is composed of 300 randomly chosen free-form questions and 151 corresponding paraphrased questions. The VQA-RAD training set is the remainder of the dataset. We conducted a manual review to calculate simple accuracies for each question type and use several accepted metrics to calculate overall performance for each model. For the manual review, we judge the predicted answers of the models to the gold standard VQA-RAD test. In addition to exact match, a full point is given for predicted answers that appropriately answer the original question. For example, “right lung” and “right upper lobe” are correct answers for the question “where is the lesion?” since the question does not indicate a degree of specification. Half a point is awarded for answers that incorporate part of the answer. For example, “right lung” would be given half a point for “which lobe is the lesion in?” where the correct answer is “right upper lobe”.

We employ multiple overall scoring methods to demonstrate limitations of current metrics and need for newer metrics to evaluate VQA in medicine. Simple accuracy is calculated as the number of correct answers over total answers. Mean accuracy is the average of accuracy of the question types, similar to TDIUC dataset^12^. The BLEU^20^ method, a commonly used metric for evaluating machine translation, measures the difference based on number of different words used. Following the ImageCLEF-Med methods, we pre-process the human and machine answers by converting answers to lower-case, removing punctuation, tokenizing individual words, and removing stop words using an English list of 172 words.

### Algorithmic validation

As might be expected, the models that best predicted VQA-RAD test set are trained on VQA-RAD training alone or partially. For closed-ended questions, MCB_RAD had slightly better simple accuracy of 60.6%, while SAN_RAD had a slightly better mean accuracy at 54.6%. This performance is comparable to the MCB and SAN models trained and tested on VQA1.0 dataset which had reported 64.2%^16^ and 58.9%^17^ accuracies for open and closed ended public domain questions. Predicting open-ended VQA-RAD questions, the models performed much lower with the MCB_RAD scoring the best at 25.4% simple accuracy and 19.3% mean accuracy. The contrast between open and closed-ended questions suggests that these models are currently still guessing and may require more data. Potential improvements can come from learning medical terminology and jargon, greater quantity and diversity of questions, and extracting image features from radiological images as opposed to public domain.

Manual evaluation of the accuracy of automatic answers to naturally occurring questions in the VQA-RAD test set is shown in Tables 2 and 3.

In medicine, seemingly small changes in wording can result in very different questions and answers. The opposite also holds true, the same idea can be written in simple or complicated phrasing. The VQA-RAD test set contains 151 matched pairs of free-form and paraphrased questions. We analyzed the MCB_RAD model to better understand the variance in predicted answers for similarly written questions. We observed 48 pairs (31.8%) had differing behavior, Table 4 shows several select pairs. Medical terminology, grammatical variations, and clinical jargon present challenges for algorithms. Paraphrasing helps understand what VQA algorithms are learning and whether those rules are aligned or need some degree of supervision. In addition, paraphrasing gives insight into the natural language of clinicians and what they consider to be similar or different concepts.

Overall scores may be useful for picking a winner of a challenge, but they may also bias towards algorithms that can provide answers that are more common. In medicine, the best answer is the most clinically relevant, not necessarily the most common. Calculating accuracies for different question types can highlight unique features of algorithm designs. For example, the SAN_RAD model scored high for open and closed ended size questions suggesting for this type of task, the SAN design might have certain advantages over the MCB design. However, further refinement is needed to understand these differences, as it is not clear if the SAN model is able to better recognize image regions of interest or is better at answering questions.

VQA-RAD demonstrates the value of natural questions for the specialized medical domain. Models without VQA-RAD training scored the same or lower than the all ‘yes’ or ‘no’ baselines, suggesting they were no better than guessing yes/no as answers. While the models may have been able to recognize yes/no structured questions, they lacked image features and vocabulary. For example, we observed that the MCB model trained on VQA1.0 dataset predicted many open-ended questions with “face”, “clock”, or “banana” as answers.

Analysis using BLEU scoring shows the need for improvement to metrics measuring visual tasks in medicine. We observe an opposite pattern between BLEU and manually judged accuracies where all ‘no’ resulted in better scores. BLEU penalizes answers with varying lengths, which favors the short one-word VQA-RAD answers and does not consider semantic similarity. While BLEU may be appropriate for certain machine learning techniques, it is not useful for medical VQA where there are many ways to phrase an answer and semantics are more valuable than exact wording. Using VQA-RAD, improved metrics can be developed that are aligned with the task of clinical care.

## Usage Notes

To ensure that all researchers understand how to use the data, we provided instructions in the Readme file in the Open Science Framework (Data Citation 1).

## Additional Information

**How to cite this article**: Lau, J. J. *et al*. A dataset of clinically generated visual questions and answers about radiology images. *Sci. Data*. 5:180251 doi: 10.1038/sdata.2018.251 (2018).

**Publisher’s note**: Springer Nature remains neutral with regard to jurisdictional claims in published maps and institutional affiliations.

## Supplementary Material



## Figures and Tables

**Table 1:** Naturally occurring question and answer types about radiology images.

Question Type	Description
**Modality**	How an image is taken – CT, x-ray, T2 weighted MRI, etc.
**Plane**	Orientation of an image slicing through the body – axial, sagittal, coronal
**Organ System**	Categorization that connects anatomical structures with pathophysiology, diagnosis, and treatment – pulmonary, cardiac, musculoskeletal system
**Abnormality**	Normalcy of an image or object. For example, “is there something wrong with the image?” or “What is abnormal about the lung?”, “Does the liver look normal?”
**Object/Condition Presence**	Objects could be normal structures like organs or body parts but could also be abnormal objects such as masses or lesions. Clinicians may refer to the presence of conditions in an image or patient – fractures, midline shift, infarction
**Positional reasoning**	position or location of an object or organ, including what side of a patient, in respect to the image borders, or relative to other objects in the image
**Color**	signal intensity including enhancement or opaqueness
**Size**	measurement of size of an object, e.g., enlargement, atrophy
**Attribute Other**	other types of description questions
**Counting**	focusing on a quantity of objects, e.g., number of lesions
**Other**	catch-all categorization for questions that do not fall into the previous categories
Answer Type	
**Close-ended**	yes/no and other limited choices. For example, “Is the mass on the left or right?”
**Open-ended**	Do not have a limited question structure and could have multiple correct answers

**Table 2:** Accuracy of the systems’ answers to the closed-ended free-form questions sub-grouped into question types.

	YES	NO	MCB_VQA1.0	MCB_CLEF	SAN_CLEF	MCB_CLEF + RAD	SAN_CLEF + RAD	MCB_RAD	SAN_RAD
ABN	33.3%	58.3%	58.3%	4.2%	29.2%	58.3%	66.7%	**79.2%**	62.5%
ATTRIB	33.3%	**66.7%**	50.0%	0%	16.7%	50.0%	50.0%	50.0%	16.7%
COLOR	50.0%	50.0%	50.0%	0%	50.0%	50.0%	50.0%	50.0%	**100.0%**
COUNT	0%	**50.0%**	0%	0%	**50.0%**	0%	0%	0%	0%
MODALITY	33.3%	46.7%	46.7%	0%	40.0%	**66.7%**	60.0%	60.0%	**66.7%**
ORGAN	100.0%	0%	100.0%	0%	0%	50.0%	100.0%	100.0%	100.0%
OTHER	**55.6%**	11.1%	33.3%	0%	11.1%	44.4%	22.2%	33.3%	11.1%
PLANE	41.7%	41.7%	25.0%	0%	50.0%	50.0%	**58.3%**	41.7%	50.0%
POS	33.3%	0%	33.3%	0%	0%	**66.7%**	33.3%	**66.7%**	66.7%
PRES	41.8%	58.2%	41.8%	40.5%	50.6%	60.8%	62.0%	**65.8%**	58.2%
SIZE	57.7%	34.6%	50.0%	7.7%	42.3%	57.7%	61.5%	50.0%	**69.2%**
**Simple Acc**	42.8%	48.9%	44.4%	19.4%	41.1%	57.8%	58.9%	**60.6%**	57.2%
**Mean Acc**	43.6%	37.9%	44.4%	4.8%	30.9%	50.4%	51.3%	54.2%	**54.6%**
**BLEU**	0.804	**0.832**	0.812	0.003	0.020	0.167	0.167	0.168	0.622
Overall, MCB_RAD and SAN_RAD performed the best. MCB_VQA1.0 shows that one of the baseline systems is not better than all yes/no baselines.									

**Table 3:** Accuracy of the systems’ answers to the open-ended free-form questions sub-grouped into questions types.

	MCB_VQA1.0	MCB_CLEF	SAN_CLEF	MCB_CLEF + RAD	SAN_CLEF + RAD	MCB_RAD	SAN_RAD
ABN	0%	0%	**5.6%**	0%	0%	0%	0%
ATTRIB	0%	0%	0%	0%	0%	0%	0%
COLOR	0%	0%	0%	0%	0%	0%	0%
COUNT	0%	0%	0%	0%	0%	0%	0%
MODALITY	0%	32.1%	0%	71.4%	50.0%	**78.6%**	57.1%
ORGAN	0%	7.1%	0%	14.3%	0%	**28.6%**	14.3%
OTHER	0%	10.0%	0%	**20.0%**	10.0%	10.0%	10.0%
PLANE	0%	0%	0%	**81.8%**	72.7%	72.7%	**81.8%**
POS	5.9%	2.9%	4.4%	16.2%	**30.9%**	22.1%	25.0%
PRES	1.7%	3.4%	0%	0.0%	**6.9%**	0%	0%
SIZE	0%	0%	0%	0.0%	0%	0%	**16.7%**
**Simple Acc**	0%	6.7%	1.7%	23.8%	24.6%	**25.4%**	24.2%
**Mean Acc**	0.7%	5.1%	0.9%	18.5%	15.5%	**19.3%**	18.6%
**BLEU**	**0.1047**	0.0058	0.0113	0.0031	0.0339	0.0308	0.0576
Overall, modality and plane questions are predicted well. Deficiencies are seen in other areas like Abnormality, Color, and Counting. MCB_VQA1.0 shows how a baseline system’s BLEU scores disagree with manual judgments.							

**Table 4:** Effect of paraphrasing on models.

Question	Answer (GS)	Answer (MCB_RAD)
Is the heart enlarged?	Yes	yes
Is cardiomegaly present?	Yes	no
The image is taken in what plane?	axial	dwi
What plane is the above image acquired in?	axial	axial
Is this a CT or an MRI?	MRI	ct
Was a CT or MRI used to take the above image?	MRI	t2 weighted
Does the liver show an enhancing mass or lesion?	No	normal
Is there an enhancing lesion in the liver?	No	no
What are the hyperdense lesions noted at the edges of the aorta?	Calcified atherosclerosis	calcifications
What are the hyperintensities surrounding the aorta?	Calcified atherosclerosis	ribs
Sample of free-form and paraphrased questions that resulted in differing predicted answers from models. MCB model is trained on MCB_RAD. GS represents gold standard answer generated by clinical annotators.
